# Variant isoforms of CD44 involves acquisition of chemoresistance to cisplatin and has potential as a novel indicator for identifying a cisplatin-resistant population in urothelial cancer

**DOI:** 10.1186/s12885-018-3988-3

**Published:** 2018-01-31

**Authors:** Masayuki Hagiwara, Eiji Kikuchi, Nobuyuki Tanaka, Takeo Kosaka, Shuji Mikami, Hideyuki Saya, Mototsugu Oya

**Affiliations:** 1 Department of Urology, Keio University School of Medicine, 35 Shinanomachi, Shinjuku-ku, Tokyo, 160-8582 Japan; 2 Division of Diagnostic Pathology, Keio University School of Medicine, Tokyo, Japan; 3 Division of Gene Regulation, Institute for Advanced Medical Research, Keio University School of Medicine, Tokyo, Japan

**Keywords:** cisplatin, chemoresistance, CD44, variant isoform, xCT

## Abstract

**Background:**

Cisplatin is the most commonly used chemotherapeutic agent in the treatment of patients with metastatic and/or recurrent urothelial cancer. However, the effectiveness of these treatments is severely limited due to the development of cisplatin resistance. Cancer stem cells have been documented as one of the key hypotheses involved in chemoresistance. CD44v8–10 has been identified as one of the new cancer stem cells markers and was recently shown to enhance the antioxidant system by interaction with xCT, a subunit of the cystine transporter modulating intracellular glutathione synthesis. The aim of the present study was to investigate the clinical role of CD44v8–10 and the molecular mechanism underlying the acquisition of cisplatin resistance through CD44v8–10 in urothelial cancer**.**

**Methods:**

We analyzed the clinical significance of the immunohistochemical CD44v9 expression, which detects the immunogen of human CD44v8–10, in 77 urothelial cancer patients treated with cisplatin-based systemic chemotherapy for recurrence and/or metastasis. We then evaluated the biological role of CD44v8–10 in the acquisition of cisplatin resistance using the urothelial cancer cell lines, T24 and T24PR, which were generated to acquire resistance to cisplatin.

**Results:**

The 5-year cancer-specific survival rate was significantly lower in the CD44v9-positive group than in the CD44v9-negative group (*P* = 0.008). Multivariate analyses revealed that CD44v9 positivity was an independent risk factor of cancer-specific survival (*P* = 0.024, hazard ratio = 5.16) in urothelial cancer patients who had recurrence and/or metastasis and received cisplatin-based chemotherapy. The expression of CD44v8–10 and xCT was stronger in T24PR cells than in T24 cells. The amount of intracellular glutathione was significantly higher in T24PR cells than in T24 cells (*p* < 0.001), and intracellular reactive oxygen species production by cisplatin was lower in T24PR cells than in T24 cells. Furthermore, the knockdown of CD44v8–10 by siRNA led to the recovery of cisplatin sensitivity in T24PR cells.

**Conclusions:**

CD44v9 in tumor specimens has potential as a novel indicator for identifying a cisplatin-chemoresistant population among urothelial cancer patients. CD44v8–10 contributes to reactive oxygen species defenses, which are involved in chemoresistance, by promoting the function of xCT, which adjusts the synthesis of glutathione.

## Background

Urothelial cancer (UC) is one of the most aggressive epithelial tumors and remains extremely challenging to treat in advanced stages [1, 2]. Surgical interventions for localized or locally advanced UC represent the most successful treatment option; however, recurrence of the disease is very common due to early systemic dissemination. Cisplatin (CDDP) is the most commonly used chemotherapeutic agent in the treatment of patients with metastatic and/or recurrent UC. Although most of these patients show good initial responses to CDDP-based combination chemotherapy, the effectiveness of these treatments is severely limited due to the development of CDDP resistance [3, 4]. Despite recent advances, only a limited number of new chemotherapeutic agents have been developed for advanced UC, and CDDP is still regarded as the key agent against metastatic and/or recurrent UC. Therefore, the mechanisms responsible for the acquisition of resistance to CDDP need to be elucidated in more detail in order to overcome this resistance.

Although the specific mechanisms involved in the development of chemotherapeutic resistance are not fully understood, it is recognized as a multifactorial process [5]. Cancer stem cells (CSCs) have been documented as one of the key hypotheses involved in the development of chemoresistance by various types of cancers [6, 7]. CD44 has been identified as one of the major cell surface markers associated with CSCs in many types of solid tumors including breast, colon, pancreatic, and prostate cancers [8–11]. CD44 exists in numerous variant isoforms generated through the alternative mRNA splicing of different combinations of 10 exons (v1–10) [12], and the variant isoforms of CD44 containing v8-v10 (CD44v8–10) have been identified as new cell surface markers for CSCs [13–18]. We previously reported the clinical and prognostic significance of CD44v9 expression, which detects the immunogen of human CD44v8–10, in upper tract urothelial cancer (UTUC) patients who underwent surgery [19]. CD44v8–10 was recently considered to enhance the antioxidant system by interaction with xCT contributes to CSCs features, including chemotherapeutic resistance [13, 16]. xCT is known as a subunit of the cystine transporter, and modulate the function of the cystine transporter. xCT has been reported to mediate intracellular glutathione (GSH) synthesis through the uptake of cystine, and contributes to the suppression of reactive oxygen species (ROS) production mediated by various types of chemotherapeutic agents [20, 21].

In the present study, we evaluated 1) the relationship between CD44v9 expression and cancer-specific survival (CSS) in UC patients with recurrence and/or metastasis after radical surgery and received CDDP-based chemotherapy in order to reveal the clinical role of CD44v9 expression in the development of chemoresistance in these patients, 2) changes in CDDP chemosensitivity and CD44v8–10 expression in a T24 platinum-resistant (T24PR) cell line established as an acquired platinum-resistant subline of T24 cells [22], and 3) the molecular mechanisms by which CD44v8–10 leads to the acquisition of CDDP resistance in T24PR cells.

## Methods

### Immunohistochemical evaluation of CD44v9 in UC patients treated with CDDP-based chemotherapy

After obtaining Institutional Review Board approval, the medical records of patients who underwent surgery for UC between 1990 and 2007 at Keio University Hospital were retrospectively reviewed. We identified 182 patients who had been surgically treated for pT2≤ invasive UC of either UTUC or bladder cancer. Patients who received chemotherapy or radiation therapy before radical surgery, and those with distant metastasis at the time of their diagnosis were excluded from our study. Seventy-seven patients were treated with CDDP-based systemic chemotherapy for recurrent and/or metastatic UC. The mean age of the entire cohort was 68 years (range, 40 to 89 years). Males accounted for 70.1% (54 patients) and females 29.9% (23 patients). During the mean follow-up period of 45 months, 53 patients (68.8%) died of the disease. Fifty-eight patients (75.3%) with UTUC underwent radical nephroureterectomy with removal of the bladder cuff and 19 patients (24.7%) with invasive bladder tumors underwent total cystectomy. In patients with bladder cancer, standard lymphadenectomy, including obturator, internal iliac, and external iliac lymph nodes, was performed up to the lower third of the common iliac arteries. Regional lymphadenectomy was generally performed on UTUC patients with suspicious lymph nodes on preoperative axial imaging or with adenopathies detected during intraoperative examinations. Adjuvant chemotherapy was administered to 34 patients (44.2%). Patients with pT3/4 tumors or lymph node metastasis were generally recommended to receive adjuvant CDDP-based chemotherapy following surgery in our institution during the study period. Postoperative adjuvant radiotherapy regimens were not routinely used. Patients were followed postoperatively with urinary cytology every 3 months for 2 years and every 6 months thereafter. Computed tomography or magnetic resonance imaging was performed every 6 months for 5 years and annually thereafter. Cystoscopy was also performed for UTUC patients every 6 months for 5 years and annually thereafter. Elective bone scans and chest computed tomography were performed when clinically indicated. The cause of death was determined by the attending physicians.

All surgical specimens were fixed in 10% formalin and embedded in paraffin. All slides were re-reviewed by genitourinary pathologists, and were histologically confirmed to be UC. Tumors were staged according to the American Joint Committee on the Cancer-Union Internationale Contre le Cancer TNM classification [23]. Tumor grading was assessed according to the 1998 WHO/International Society of Urologic Pathology consensus classification [24]. Lymphovascular invasion was defined as the presence of tumor cells within an endothelium-lined space without underlying muscular walls.

We carried out immunohistochemical staining for human CD44v8–10. Four-micrometer-thick sections of formalin-fixed and paraffin-embedded material were analyzed. These sections were deparaffinized in xylene and rehydrated in graded alcohols and distilled water. After antigen retrieval with citric acid (pH 6.0) for 10 min at 105 °C, endogenous peroxidase activity was blocked with 1% hydrogen peroxide for 20 min followed by washing with distilled water. In order to bind non-specific antigens, the sections were incubated for 15 min at room temperature with 6% skim milk in PBS. The sections were incubated at 4 °C overnight with an anti-CD44v9 rat monoclonal antibody, which detects the immunogen of human CD44v8–10 (1:5000 dilation, Cosmo Bio, Tokyo, Japan). After washing with PBS, tissue sections were incubated with secondary antibody against rat primary antibody (Histofine Simple Stain MAX PO (Rat), Nichirei Biosciences, Tokyo, Japan) for 30 min. An immunoreaction was detected using the avidin-biotin complex peroxidase method. Color was developed with 3, 30-diaminobenzamine tetrahydrochloride in 50 mmol/L Tris-HCl (pH 7.5) containing 0.005% hydrogen peroxide. Sections were counterstained with hematoxylin. Negative control was carried out by omitting the primary antibody, and gastric cancer sections, which had been evaluated and considered positive expression for CD44v9 in a previous report [25], were used as positive control for CD44v9.

In order to evaluate CD44v9 staining, cancer cells with positive staining in the cell membrane were counted in at least 10 representative fields, and the mean percentage of positive cancer cells was estimated. We used the proportion of positive cells of CD44v9 expression as a scoring system. The density of CD44v9 in tumor cells was scored as the average proportion of detectable immunoreactions in 10 representative fields (range, 0%–100%) for each tumor. This scoring system assessing only the proportion of positive cells for CD44v9 expression was also used in previous reports in gastric cancer and UTUC [19, 25], and we assigned patients to a CD44v9-positive group or a CD44v9-negative group based on a cut-off level of 5% in CD44v9 density, same as previous report indicated prognostic significance of CD44v9 immunohistochemical expression in UTUC patients [19]. Two authors blinded to patient data independently evaluated immunoreactivity for CD44v9 staining.

### Immunofluorescence

To measure immunofluorescence, 2 × 10^4^ cells were seeded on 14 mm coverslips in 8-well plates. After 24 h the cells were washed with PBS, fixed in 4% paraformaldehyde-PBS for 20 min at room temperature, and then permeabilized in cold PBS with 0.2% TritonTM X-100 for 10 min at room temperature. Blocking was done with PBS, 3% bovine serum albumin, 0.1% saponin and 0.02% azide for 40 min at room temperature. The slides were then incubated with primary antibody (anti-CD44v9 rat monoclonal antibody, 1:300 dilution) for 1 h at room temperature and thereafter with anti-rat Alexa 555 antibody (dilution 1:500). Coverslips were mounted on glass slides with 4′, 6-diamidino-2-phenylindole containing Vectashield® mounting medium and visualized by confocal microscopy.

### Cell culture and chemicals

T24, a human bladder cancer cell line, was obtained from the ATCC (ATCC HTB-4). Two UC cell lines (T24 and T24PR) were routinely maintained in RPMI-1640 (Invitrogen, Carlsbad, CA) with 10% fetal bovine serum at 37 °C in a humidified 5% CO_2_ atmosphere. The T24 cell line was obtained from the American Type Culture Collection more than 1 year ago from each experiment. The T24PR cell line was generated to acquire resistance to CDDP from T24 cells in our laboratory. T24 cells were grown and passaged upon reaching confluence in medium containing CDDP over a 6-month period in order to develop platinum resistance, and the concentration of CDDP was then increased up to 3 μM. Further examinations were performed after 6 months without CDDP exposure in order to completely eliminate the influences of stress caused by CDDP on T24PR cells. Although long-term subculture changed the features of cell lines, we needed the long-term subculture to perform experiments in the present study. In order to exclude these changes and focus on the changes in acquisition of CDDP-resistance, we also cultured T24 cells in long term same as T24PR cells in medium without CDDP, as control, and compared these cells. Therefore, the cell lines using this experiment have not been tested and authenticated immediately before the examinations. CDDP was purchased from Sigma-Aldrich (Atlanta, GA).

### Cell extracts and western blot analysis

Whole cell extracts were obtained using radioimmunoprecipitation assay buffer (50 mmol/L Tris-HCL (pH 7.5), 150 mmol/L NaCl, 1% NP-40, 0.5% deoxycholate, and 0.1% SDS) containing protease inhibitors. In the Western blot analysis, 50 mg of total protein from each sample was loaded on 12.5% SDS-polyacrylamide gels. Immunoblotting was also performed according to a standard method. Proteins were transferred onto a polyvinylidene difluoride membrane in blocking solution (5% non-fat dry milk in TBS containing 0.1% Tween 20). The primary antibody for the cytoplasmic region of CD44 was a rabbit polyclonal antibody (1:1000 dilution; TransGenic, Kobe, Japan), that for xCT was a rabbit polyclonal antibody (1:1000 dilution; Abcam, Cambridge, GA), and that for β-actin was a mouse monoclonal antibody (1:1000 dilution; Sigma-Aldrich, Atlanta, GA). After washing, the membranes were incubated at room temperature for 1 h linked with a peroxidase secondary antibody (Dako, Denmark), and signals were detected and the intensity was quantified using the LAS4000 Image Analysis System (GE, Fairfield, CT).

### Intracellular GSH and ROS measurements

Regarding cellular GSH measurements, 1 × 10^4^ T24 or T24PR cells in 100 μL of culture medium were plated on each well of a 96-multiwell white plate, allowed to attach for 24 h, and each well was then washed three times with PBS. Following the addition of 100 μL of GSH-Glo Reagent (Promega Corp., Madison, WI) at room temperature for 30 min, 100 μL of the luciferin detection reagent was added at room temperature for an additional 15 min. The luminescence intensity of each well was recorded on a GloMax™ 96 Microplate Luminometer (Promega Corp., Madison, WI).

In cellular ROS measurements, 1 × 10^4^ T24 or T24PR cells in 100 μL of culture medium were plated on each well of a 96-multiwell white plate for 24 h and were treated with various concentrations of CDDP for 24 h. Cellular H_2_O_2_ was assessed by adding 20 μL of the ROS-Glo H_2_O_2_ substrate (Promega Corp., Madison, WI) to each well, which were then left standing at 37 °C for 2 h in a humidified 5% CO_2_ atmosphere. A 100 μL aliquot of ROS-Glo detection solution was added to the resulting mixture and incubated at room temperature for 20 min. The luminescence intensity of each well was recorded on a GloMax™ 96 Microplate Luminometer.

### siRNA transfection

CD44v8–10 expression was transiently down-regulated using the following predesigned siRNA duplexes directed against CD44v8–10 (CD44v8–10 siRNA #1 and #2) [26]. siRNAs specific for CD44v8–10 and non-targeting control (NTC) siRNA were synthesized from Sigma-Aldrich (Atlanta, GA). The sequences of siRNA duplexes for CD44v8–10 and NTC were as follows: CD44v8–10 siRNA #1, sense, 5’-GGAAGAAGAUAAAGACCAUUU-3′, antisense, 5’-AUGGUCUUUAUCUUCCUU-3; CD44v8–10 siRNA #2, sense, 5’-CUACUUUACUGGAAGGUUAUU-3′, antisense, 5’-UAACCUUCCAGUAAAGUAGUU-3; control siRNA, sense, 5’-rCrArAUrAUUrGrArGUrArGrCrGUUrCU-3′, antisense, 5’-rArGrArArCrGrCUrArCUrCrArAUrAUUrG-3. T24PR cells were transiently transfected with 10 nmol of CD44v8–10 siRNA #1, CD44v8–10 siRNA #2, or NTC for 48 h with the use of Lipofectamine RNAi MAX reagent (Invitrogen, San Diego, CA).

### Cell viability assay

T24, T24PR, or T24PR cells transfected with siRNA were plated on 96-well plates, allowed to attach for 24 h, and then incubated for 48 h with various concentrations of CDDP in order to investigate the sensitivity of the cell lines to CDDP. At the end of the incubation period, water-soluble tetrazolium reagents (Takara Bio Inc., Shiga, Japan) were added to each well and incubated for 1 h. Cell viability was estimated by colorimetry, with color intensity being read on a plate reader at 570 nm.

### Statistical analysis

The relationships between CD44v9 and clinicopathological features were assessed using the χ^2^ test. CSS were calculated by the Kaplan-Meier method and analyzed by the log-rank test. Cox proportional hazards regression analysis with stepwise forward selection was used to assess prognostic indicators including age, gender, tumor location, tumor grade, pathological T stage, lymphovascular invasion, lymph node metastasis, and CD44v9 expression for survival. The significance of differences between the two groups in the in vitro study was assessed with the Mann-Whitney *U* test. The level of significance was set at *P* < 0.05. These analyses were performed with the SPSS Version 21.0 statistical software package.

## Results

### The clinical role of CD44v9 expression in UC human samples

#### Relationships between CD44v9 expression and clinicopathological features in recurrent/metastatic UC treated with CDDP-based chemotherapy

**Fig. 1:**
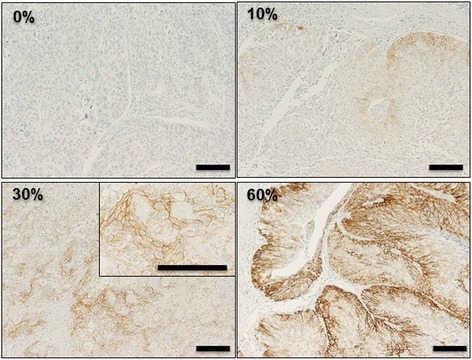
Representative immunostaining of UC tissue for CD44v9 in patients with CD44v9 densities of 0%, 10%, 30%, and 60%. In CD44v9-positive tumors, CD44v9 was expressed in the epithelium of tumor glands with a heterogeneous expression pattern. At 400× magnification, the expression of CD44v9 was detected along the cell membrane. The bar indicates 200 μm

In order to elucidate the biological significance of CD44v8–10 in UC, we examined the immunohistochemical expression of CD44v9, which detects the immunogen of human CD44v8–10. Representative CD44v9 immunohistochemical staining is shown in Fig. 1. In CD44v9-positive tumors, CD44v9 was expressed in the epithelium of tumor glands with a heterogeneous expression pattern. Under a high-power field, the expression of CD44v9 was detected along the tumor cell membrane. In sections of CD44v9-positive tumor specimens, no protein expression of CD44v9 was observed in cells of normal urothelial epithelium. Patients were then allocated into the CD44v-positive group (*n* = 64, 83.1%) or CD44v-negative group (*n* = 13, 16.9%) based on a cut-off level of 5% in CD44v9 density, as reported previously [19]. Table 1 shows the relationships between clinicopathological parameters and CD44v9 expression in our study population. Patients with CD44v9-positive expression had a significantly higher incidence of pT3/4 tumors.

**Table 1:** Relationship between CD44v9 expression and clinicopathological characteristics in invasive UC patients treated with CDDP-based chemotherapy against recurrence and/or metastasis after surgery

	CD44v9		
	Negative	Positive	*p*-value
Age			
< 68	5	29	0.650
≥ 68	8	35	
Gender			
male	9	45	0.938
female	4	19	
Tumor location			
upper tract	9	49	0.576
bladder	4	15	
Tumor grade			
G1/2	3	4	0.054
G3	10	60	
Pathological T stage			
pT2	5	7	0.013
pT3/4	8	57	
Lymphovascular invasion			
negative	5	15	0.260
positive	8	49	
Lymph node metastasis			
pNx or pN0	10	54	0.722
pN1, 2	3	10	

#### Prognostic significance of CD44v9 expression in recurrent/metastatic UC treated with CDDP-based chemotherapy

**Fig. 2:**
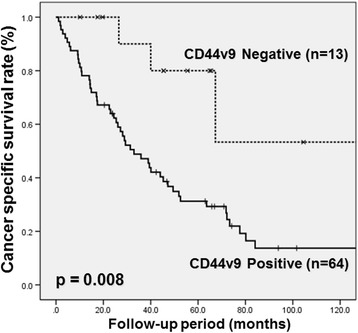
Kaplan-Meier curve of cancer-specific survival in 77 UC patients treated with chemotherapy against recurrence and/or metastasis according to CD44v9 expression. Patients were allocated into the CD44v9-positive group (*N* = 64) or CD44v9-negative group (*N* = 13) based on a cut-off level of 5% in CD44v9 density. Cancer-specific survival rates were significantly lower in the CD44v9-positive group than in the CD44v9-negative group (*p* = 0.008 by log-rank test)

The Kaplan-Meier curve demonstrated that CSS rate was significantly lower in the CD44v9-positive group than in the CD44v9-negative group in UC patients who were treated with CDDP-based chemotherapy (Fig. 2). The 3- and 5-year CSS rates were 47.1% and 31.2% in the CD44v9-positive group and 90.0% and 80.1% in the CD44v9-negative group (*p* = 0.008), respectively. Univariate and multivariate Cox regression analysis were performed in order to identify risk factors for cancer-specific mortality (Table 2). The univariate analysis identified tumor grade G3 (*p* = 0.025) and CD44v9 expression (p = 0.008) as significant risk factors for cancer-specific mortality. The multivariate analysis showed that CD44v9 expression (*p* = 0.024, Hazard ratio = 5.16) was an independently associated with cancer-specific mortality.

**Table 2:** Univariate and multivariate Cox regression analyses predicting significant risk factors for cancer-specific mortality in invasive UC patients treated with CDDP-based chemotherapy against recurrence and/or metastasis

Characteristic	Univariate	Multivariate	
	*P* value	HR (95%CI)	P value
Age (< 68 years vs. ≥ 68 years)	0.499		
Gender (Male vs. Female)	0.593		
Location (Upper tract vs. Bladder)	0.614		
Grade (G1/2 vs. G3)	0.025		0.090
Pathological T stage (pT2 vs. pT3/4)	0.246		
Lymphovascular invasion (Negative vs. Positive)	0.286		
Lymph node status (pNx or pN0 vs. pN1, 2)	0.661		
CD44v9 (Negative vs. Positive)	0.008	5.16 (1.24–21.52)	0.024

### Relationship between CD44v8–10 and acquired CDDP chemoresistance evaluated in the in vitro study using UC cell lines

#### Protein expression of CD44v8–10 and xCT, and cytotoxic effects against CDDP in T24 and T24PR cells

**Fig. 3:**
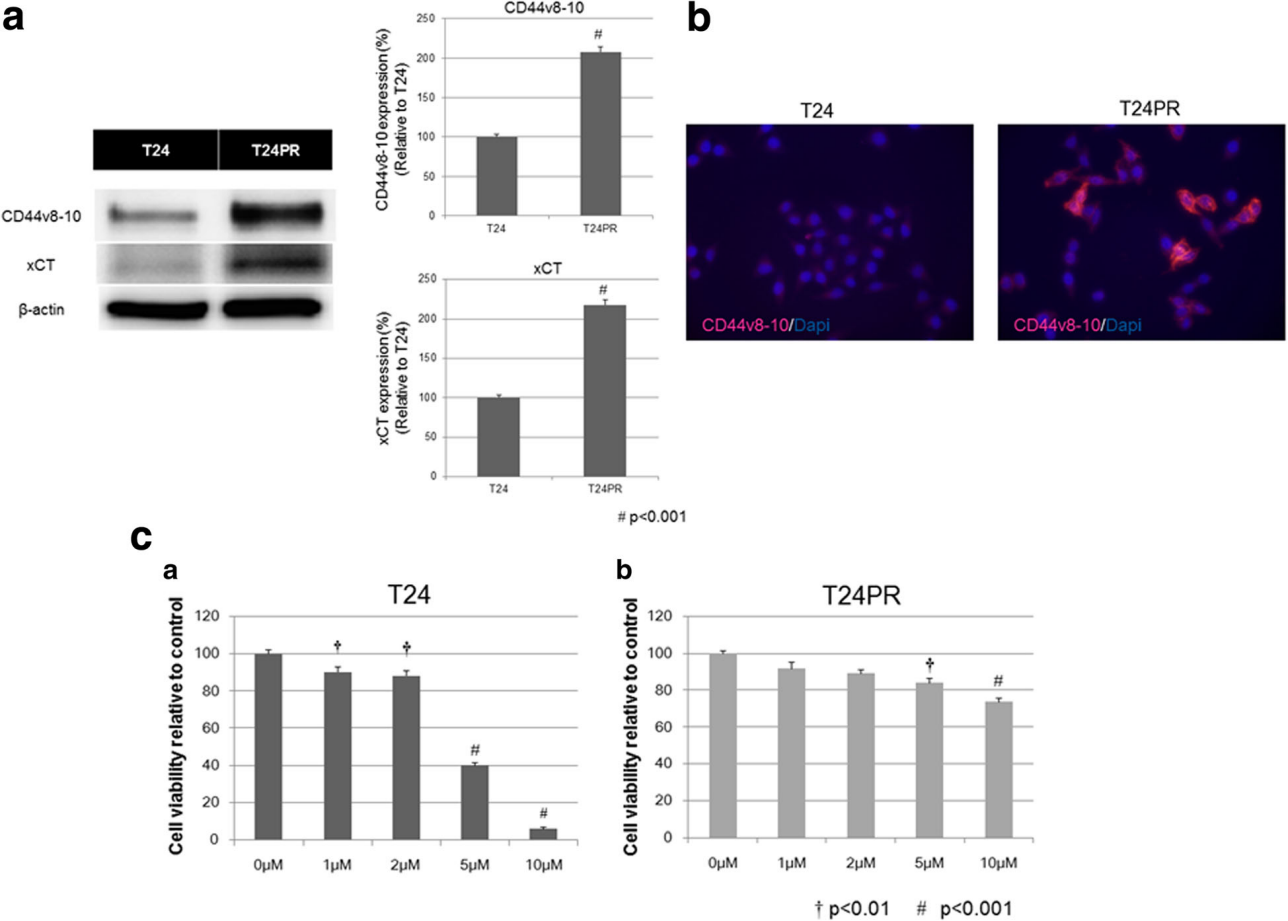
Protein expression of CD44v8–10 and xCT, and cytotoxic effects of CDDP in T24 and T24PR cells. **a** Western blot analysis of protein expression of CD44v8–10 and xCT, a subunit of the cystine transporter, in T24 cells and T24PR cells which was generated to acquire resistance to CDDP from T24 cells in our laboratory and these signal intensities. Left panel) Western blot analysis shows the stronger expression of CD44v8–10 and xCT proteins in T24PR cells as compared to those in T24 cells. Right panel) The signal intensities of the protein expression of CD44v8–10 and xCT in T24PR cells were significantly higher than those in T24 cells (*p* < 0.001 for both protein expression). **b** Immunofluorescence staining of CD44v8–10 expression in T24 and T24PR cells. Immunofluorescence staining shows CD44v8–10 protein expression in T24PR cells was stronger than that in T24 cells. **c** Cell viability relative to control at various concentrations of CDDP (0 to 10 μM) in (a) T24 cells and (b) T24PR cells. A cell viability assay showed that the IC50 of CDDP in T24PR cells was about 5-fold higher than that in T24 cells (T24: 4.1 μM, T24PR: 19.3 μM). †; *p* < 0.01, #; *p* < 0.001, compared with vehicle control (without CDDP exposure)

In order to investigate the involvement of CD44v8–10 in the acquisition of CDDP resistance by UC cells, we analyzed the expression of CD44v8–10 at the protein level in T24 and T24PR cells using Western blot analysis (Fig. 3a). The signal intensity of CD44v8–10 protein expression was stronger in T24PR cells that acquired resistance to CDDP than in their corresponding parent cells, T24 (*p* < 0.001). We also analyzed the expression of xCT, which is considered to be stabilized by an interaction with CD44v8–10. The signal intensity of xCT protein expression was also stronger in T24PR cells than in T24 cells (p < 0.001). In addition, we confirmed the stronger CD44v8–10 expression in T24PR cells compared with T24 cells using immunofluorescence staining (Fig. 3b). Significant cytotoxic reduction was observed in T24 cells treated with a concentration of 1μM or higher of CDDP as compared to those treated with the vehicle control (Fig. 3c). However, significant cytotoxic reduction was only observed in T24PR cells treated with CDDP at a concentration of 5μM or higher as compared to those treated with the vehicle control. The IC_50_ of CDDP in T24PR cells was 19.3μM, which was almost 5-fold higher than that in T24 cells (4.1μM).

#### Intracellular GSH levels in T24 and T24PR cells and their ROS production by CDDP

**Fig. 4:**
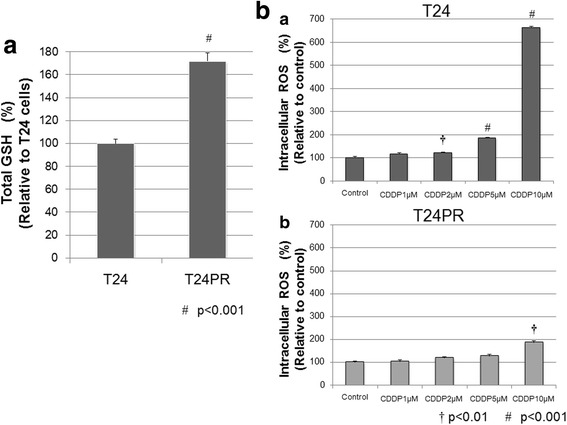
Intracellular GSH levels in T24 and T24PR cells and their ROS production by CDDP. **a** Intracellular GSH levels in T24 and T24PR cells. Intracellular GSH levels were significantly higher in T24PR cells than in T24 cells (#; *p* < 0.001). **b** Intracellular ROS production in T24 and T24PR cells after exposure to various concentrations of CDDP (0 to 10 μM). In T24 cells, significant ROS production was observed at CDDP concentrations of 2 μM or higher as compared to vehicle control. In T24PR cells, significant ROS production was observed only at a CDDP concentration of 10 μM. †; *p* < 0.01, #; *p* < 0.001

In order to investigate the role of CD44v8–10 in the regulation of cellular antioxidant capacity through xCT in UC cells, we measured intracellular GSH levels and ROS production by CDDP in T24 and T24PR cells. Intracellular GSH levels were significantly higher in T24PR cells than in T24 cells (*p* < 0.001, Fig. 4a). ROS production by T24 cells exposed to CDDP increased in a dose-dependent manner (Fig. 4b). On the other hand, significant changes in ROS production were not observed in T24PR cells exposed to CDDP up to a concentration of 10μM.

#### CD44v8–10 knockdown by siRNA increases the sensitivity of T24PR cells to CDDP

**Fig. 5:**
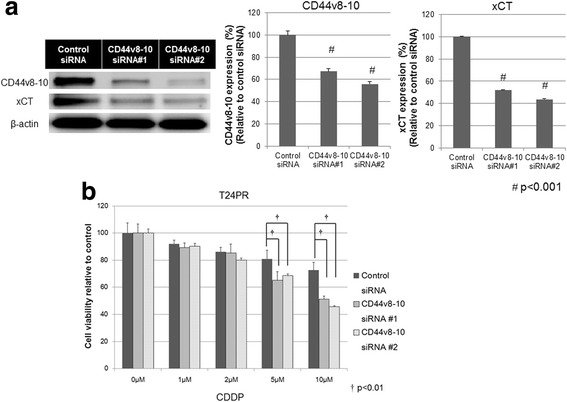
Knockdown for CD44v8–10 using siRNA increases the sensitivity of T24PR cells to CDDP. **a** Western blot analysis of protein expressions of CD44v8–10 and xCT in T24PR cells after transfection of siRNA specific for CD44v8–10 and these signal intensities. Left panel) Western blot analysis shows that the protein expressions of CD44v8–10 and xCT were reduced in T24PR cells transfected with siRNA #1 and siRNA #2 for CD44v8–10 as compared to those in T24PR cells transfected with siRNA for a non-targeting control. Right panel) The signal intensities of the protein expression of CD44v8–10 and xCT in T24PR cells transfected with siRNA #1 and siRNA #2 for CD44v8–10 were significantly lower than those in T24PR cells transfected with siRNA for a non-targeting control (#; p < 0.001 for both protein expression). **b** Cell viability relative to control at various concentrations of CDDP exposure in T24PR cells transfected with siRNA specific for CD44v8–10. After exposure to 5 μM and 10 μM CDDP, the relative cell viabilities to vehicle control in T24PR cells transfected with siRNA #1 and siRNA #2 for CD44v8–10 were significantly lower than those in T24PR cells transfected with siRNA for a non-targeting control (†; *p* < 0.01)

In order to determine whether the knockdown of CD44v8–10 expression affects CDDP resistance in T24PR cells, we evaluated the cytotoxic effects of CDDP in T24PR treated with siRNA specific for CD44v8–10. A Western blot analysis indicated that the protein expression of CD44v8–10 and xCT were reduced in T24PR cells transfected with siRNA #1 and siRNA #2 for CD44v8–10 as compared to those transfected with siRNA for NTC (Fig. 5a). After being exposed to 5μM CDDP, cell viabilities in T24PR cells transfected with siRNA#1 specific for CD44v8–10 (65.1±6.3%) and siRNA#2 specific for CD44v8–10 (68.6±1.3%) were significantly lower than that in T24PR cells transfected with siRNA for NTC (80.9±6.5%, *p* < 0.01, Fig. 5b). After being exposed to 10μM of CDDP, cell viabilities in T24PR cells transfected with siRNA#1 specific for CD44v8–10 (51.1±2.4%) and siRNA#2 specific for CD44v8–10 (45.5±0.8%) were significantly lower than that in T24PR cells transfected with siRNA for NTC (72.7±5.6%, *p* < 0.01).

## Discussion

Among the variant isoforms of the CD44 family, CD44v8–10 was recently found to contribute to CSCs features, such as tumor aggressiveness and therapeutic resistance [13–18]. Especially in gastric cancer cells, CD44v8–10 was associated with chemotherapeutic resistance through the stabilization of xCT functions by combining together on the tumor cell surface [13]. The expression of CD44v9, which detects the immunogen of human CD44v8–10, in tumor tissues has been reported as a predictive marker for a higher tumor recurrence rate and poor prognosis in several types of cancer [25, 27–29]. In addition, recent studies have indicated that CD44v9 expression in tumor specimens was one of the prognostic factors in both bladder cancer and UTUC patients [19, 30]. Despite CSCs appearing to be primarily responsible for the failure of treatments, clinical research studies have not yet addressed the involvement of CD44v9, which is one of the new CSC markers, in chemoresistance. In the present study, we retrospectively evaluated the impact of CD44v9 protein expression in tumor specimens on cancer survival in UC patients with tumor recurrence and/or metastasis after radical surgery and who were treated with CDDP-based chemotherapy. Our results revealed that patients with positive CD44v9 expression had significantly lower CSS rates and thus, CD44v9 positivity in tumor specimens was identified as an independent predictor for a poor prognosis in UC patients who received CDDP-based chemotherapy. To the best of our knowledge, this is the first study to examine the relationship between CD44v9 expression and chemoresistance in UC patients with tumor recurrence and/or metastasis.

Several recent reports showed an association of CD44 with therapeutic resistance in UC. Tatokoro et al. demonstrated that CD44-positive bladder cancer cells have greater CDDP resistance than CD44-negative cells [31]. Wu et al. reported that the staining of CD44 was significantly linked with a lower response rate chemoradiation therapy, and concluded that CD44-positive bladder cancer cells appeared more resistant to irradiation [32]. However, the detailed mechanism responsible for therapeutic resistance in CD44-positive UC cells has not yet been elucidated.

We evaluated CD44v8–10 expression levels in a T24PR cell line that acquired resistance to CDDP in order to elucidate the involvement of CD44v8–10 in the process of obtaining CDDP resistance, and found that CD44v8–10 expression levels were higher in T24PR cells than those in their parent cell line, T24. Furthermore, cytotoxicity for CDDP was almost 5-fold lower in T24PR cells than in T24 cells. These results demonstrated the close relationship between CD44v8–10 expression and acquired resistance to CDDP in UC cells We also investigated the expression level of xCT, which interacts with and is stabilized by CD44v8–10. Our results revealed that the expression of xCT was higher in T24PR cells in which CD44v8–10 expression was highly elevated. Previous studies indicated that the expression of xCT was associated with tumor recurrence and poor survival in patients with various types of solid malignancies, including colorectal cancer, hepatocellular cancer, and esophageal squamous cell cancer [33–35]. Furthermore, in ovarian cancer, the up-regulation of xCT functions has been reported as one of the mechanisms responsible for chemoresistance [36].

We then evaluated whether the knockdown of CD44v8–10 improves CDDP chemosensitivity through the suppression of xCT in T24PR cells, and found down-regulated expression of CD44v8–10 and xCT as well as the recovery of CDDP chemosensitivity in T24PR cells transfected with siRNA specific for CD44v8–10. Our results suggested that a therapeutic modality targeting the CD44v8–10-xCT-dependent antioxidant system might be one of the novel approaches to overcome CDDP resistance in UC. Previous studies reported that sulfasalazine, which is a drug used for the treatment of inflammatory bowel disease and rheumatoid arthritis, is a specific inhibitor of xCT-mediated cystine transporters [37]. Pharmacological inhibition by sulfasalazine was recently shown to selectively damage CD44v8–10-expressing gastric cancer cells, while a sulfasalazine treatment suppressed CD44v8–10-dependent chemoresistance [13]. With regard to UC, one study indicated the effectiveness of sulfasalazine in UC cells in combination with CDDP [38]. In addition, a previous clinical case report showed that a metastatic bladder cancer patient with positive CD44v9 expression in his cancer tissue had a complete response by multidisciplinary therapy including CDDP-based chemotherapy with administration of sulfasalazine for the treatment of rheumatoid arthritis [39]. These findings suggest that inhibition of the CD44v8–10-xCT-dependent antioxidant system with sulfasalazine is a promising therapeutic approach in cancer therapy.

## Conclusion

CD44v9 expression in tumor specimens has potential as a novel indicator for identifying a CDDP-chemoresistant population among surgically treated UC patients. CD44v8–10 contributes to ROS defenses, which are involved in chemoresistance, by promoting the function of xCT, which adjusts the synthesis of GSH. A therapeutic modality targeting the CD44v8–10-xCT-dependent antioxidant system may be a promising approach with which to overcome CDDP resistance in UC.

## Abbreviations

CDDP: Cisplatin
CSCs: Cancer stem cells
CSS: Cancer-specific survival
GSH: Glutathione
NTC: Non-targeting control
ROS: Reactive oxygen species
UC: Urothelial cancer
UTUC: Upper tract urothelial cancer
